# Persistent Neuroadaptations in the Expression of Genes Involved in Cholesterol Homeostasis Induced by Chronic, Voluntary Alcohol Intake in Rats

**DOI:** 10.3389/fnmol.2018.00457

**Published:** 2018-12-13

**Authors:** Josette Alsebaaly, Emilie Dugast, Laure Favot, Lydia Rabbaa Khabbaz, Marcello Solinas, Nathalie Thiriet

**Affiliations:** ^1^Laboratoire de Neurosciences Expérimentales et Cliniques, Université de Poitiers, INSERM, U-1084, Poitiers, France; ^2^Laboratoire de Pharmacologie, Pharmacie Clinique et Contrôle de Qualité des Médicaments (LPCQM), Faculty of Pharmacy, PTS, University of Saint-Joseph of Beirut, Beirut, Lebanon; ^3^CHU de Poitiers, Poitiers, France; ^4^Laboratoire Inflammation, Tissus Epithéliaux et Cytokines (LITEC), EA4331, University of Poitiers, Poitiers, France

**Keywords:** alcohol, addiction, cholesterol metabolism, gene expression, neuroadaptations

## Abstract

Alcohol use disorder (AUD) is associated with persistent adaptations in the brain that are believed to participate in the long-lasting vulnerability to relapse after abstinence. Cholesterol, the major sterol compound found in the central nervous system (CNS), plays a major role in maintenance of neuronal morphology, synaptogenesis and synaptic communication and may be involved in alcohol-induced neuroadaptations. In this study, we investigated whether alcohol consumption in a two-bottle choice paradigm followed by 3 weeks of abstinence could alter the expression of genes encoding proteins involved in cholesterol homeostasis in brain regions involved in addiction and relapse, namely the prefrontal cortex (PFC), the nucleus accumbens (NAc), the mesencephalon and the amygdala. We found that voluntary alcohol intake followed by 3 weeks of forced abstinence produces changes in the transcription of several genes encoding proteins directly involved in cholesterol synthesis such as 3-hydroxyl-3-methylglutaryl-coenzyme A (HMGCoA) reductase, farnesyl-diphosphate farnesyltransferase 1 (FDFT1) and farnesyl diphosphate synthase (FDPS) and in its regulation such as sterol regulatory element-binding factor-2 (SREBF2), in cholesterol transport such as ATP-binding cassette subfamily A member 1 (ABCA1) and in cholesterol degradation such as CYP46A1. Interestingly, these changes appeared to be region-specific and suggest that previous chronic exposure to alcohol might durably increase cholesterol metabolism in the PFC, the NAc and the mesencephalon and decrease cholesterol metabolism in the amygdala. Altogether, these results suggest that alcohol consumption leads to durable deregulations in cholesterol metabolism in key areas involved in loss of control over drug use and addiction. These long-term neuroadaptations may participate in the changes in brain structure and functioning that are responsible for the long-lasting risks of relapse to alcohol.

## Introduction

Alcohol use disorder (AUD) is a chronic relapsing brain disease that represents an important health problem and a big economic cost for our societies (Moss, 2013). AUD is characterized by episodes of intoxication, use of alcohol despite negatives consequences, compulsion to seek and consume alcohol and negative emotional states when alcohol use is stopped (Gilpin and Koob, 2008). As other drugs of abuse, alcohol produces its rewarding effects by stimulating the dopaminergic neurons of the mesocorticolimbic pathway that originates in the ventral tegmental area and projects to the nucleus accumbens (NAc), the prefrontal cortex (PFC) and the amygdala (Gessa et al., 1985; Di Chiara and Imperato, 1988). Alcohol by acting on ionotropic receptors physiologically targeted by glutamate, GABA, acetylcholine and serotonin, increases the activity of dopaminergic neurons leading to increases in dopamine levels in targeted areas (Spanagel, 2009).

Chronic exposure to alcohol produces short-term, as well as long-term neuroadaptations in the brain that could participate to AUD (Most et al., 2014). Acute withdrawal from alcohol produce many molecular, functional and behavioral alterations that result in well-defined withdrawal symptoms (i.e., tremors, risks of delirium tremens and seizures), these effects are usually short lasting (1–3 days) and disappear over time (Heilig et al., 2010). At the molecular level, acute withdrawal from chronic exposure to alcohol has been shown to potentiate glutamatergic neurotransmission by increasing the expression of NMDA receptors (NMDARs). In fact, post-mortem studies have described increased expression of the NMDA type 2B subunit in the PFC and hippocampus of human alcoholics (Zhou et al., 2011; Warden and Mayfield, 2017). These observations have also been described in animal models (for review see Roberto and Varodayan, 2017). In addition, adaptations in GABAergic neurotransmission in a region-specific manner have been described (for review see Roberto and Varodayan, 2017). Other adaptations have been shown to take place after protracted abstinence from alcohol (>3 weeks), when physical symptoms of withdrawal have disappeared and other symptoms such as elevated anxiety and dysphoria appear (Heilig et al., 2010). These molecular long-term adaptations appear to reduce the activity of the dopaminergic system which could be responsible for the negative affect symptoms associated with abstinence from alcohol and ultimately for the persistent risks of relapse to alcohol consumption (Melis et al., 2005; Volkow et al., 2007).

Recent evidence indicates that in the brain, lipids in general and sterols in particular, could play a role in neuronal adaptations. Cholesterol is the major sterol found in the central nervous system (CNS; Martin et al., 2014) and its homeostasis depends on local synthesis and turnover since it does not cross the brain blood barrier (Saeed et al., 2014). Cholesterol is mainly present in the myelin sheath formed by oligodendrocytes to insulate neurons (Dietschy and Turley, 2004; Saher and Stumpf, 2015); however neurons and other glial cells also contain large amounts of cholesterol (Martin et al., 2014). In the adult brain, cholesterol is produced in astrocytes by a multistep process called the mevalonate pathway, in which the rate limiting step is the activity of the hydroxymethylglutaryl-CoA (HMGCoA) reductase (Rozman and Monostory, 2010). Cholesterol is then exported from astrocytes after binding to lipoproteins (mainly the apolipoprotein E, called APOE; Mahley, 2016) and passage through membrane transporters, such as ATP-binding cassette subfamily A member 1 (ABCA1; Dietschy and Turley, 2004; Chen et al., 2013; Lecis and Segatto, 2014). Cholesterol is imported in neurons by binding to low density lipoprotein receptor (LDLr) and LDL-receptor like protein (Beffert et al., 2004). Cholesterol in excess is then degraded by the enzyme 24S-cholesterol hydroxylase also named CYP46A1, which is mostly expressed in neurons (Russell et al., 2009). Several sterol compounds (oxysterols, isoprenoids, neurosteroids, etc.) produced by the mevalonate pathway are known to have also their own activity in the brain (Reddy, 2010; Sun et al., 2016b; Moutinho et al., 2017). At the cellular level, cholesterol content plays a role in maintaining neuronal membrane integrity and in determining membrane permeability and fluidity (Korade and Kenworthy, 2008; Egawa et al., 2016). In addition, cholesterol has been shown to be involved in synaptogenesis, maintenance of neuronal morphology and synaptic communication (Petrov et al., 2016). Importantly, recent studies suggest that cholesterol plays a role in synaptic plasticity (Egawa et al., 2016). For example, induction of long-term potentiation (LTP) in the hippocampus produces a redistribution of intracellular cholesterol, activation of cdc42 protein which finally results in potentiation of AMPA currents (Brachet et al., 2015). In addition, cholesterol depletion inhibits synaptic transmission and synaptic plasticity and impairs NMDAR-mediated signaling and LTP (Koudinov and Koudinova, 2001; Frank et al., 2008; Korinek et al., 2015). Interestingly, synaptic plasticity processes such as LTP have been proposed to participate in addiction (Lüscher and Malenka, 2011) and therefore, changes in the cholesterol metabolism may results in altered synaptic plasticity and participate in addiction processes.

Accumulating evidences suggest that brain cholesterol homeostasis could play a role in drug addiction. First of all, a transcriptomic post-mortem study in humans found that genes involved in the synthesis and trafficking of cholesterol are overexpressed in the cortex of individuals with a history of cocaine, cannabis and phencyclidine abuse (Lehrmann et al., 2006). Second, in animal models, studying the transcriptional changes induced by alcohol exposure, McClintick et al. (2016) found that the expression of genes implicated in the cholesterol synthesis, such as HMGCoA reductase and HMGCoA synthase, is altered by exposure to alcohol in the periaqueductal gray. Finally, indirect evidence for the role of cholesterol in addiction comes from a study showing that chronic treatment with brain-penetrating statins, which are inhibitors of the HMGCoA reductase enzyme (Law and Rudnicka, 2006; Fracassi et al., 2017), reduces drug seeking for cocaine and nicotine without affecting food-seeking behavior in a rodent model of addiction (Chauvet et al., 2016). Altogether, these evidences suggest that altered cholesterol homeostasis may be a mechanism involved in addiction to drugs including alcohol.

The aim of our study was to investigate neuroadaptations in cholesterol homeostasis induced by alcohol consumption that persist after discontinuation of voluntary exposure to alcohol. We used intermittent exposure to alcohol in a two-bottle choice procedure followed by 3 weeks of abstinence. We chose this time point because it corresponds to period of protracted abstinence in rodents, a time when physical symptoms of withdrawal have disappeared (Heilig et al., 2010). These neuroadaptations are believed to be related to high risks of relapse to alcohol and to the phenomenon of incubation of craving (Pickens et al., 2011) that has been described for several drugs including alcohol (Bienkowski et al., 2004). To screen for changes in the expression of many of genes encoding proteins involved in the brain cholesterol metabolism in several brain areas, we chose to measure the mRNA levels of candidate genes by real time PCR (RT-PCR). We focused our investigation on the PFC, the NAc, the amygdala and the mesencephalon, which are known to play a major role in alcohol addiction (Volkow et al., 2016; Cooper et al., 2017). We investigated the expression of genes encoding enzymes of the mevalonate pathway [HMGCoA synthase, HMGCoA reductase, farnesyl diphosphate synthase (FDPS), farnesyl diphosphate farnesyltransferase 1 (FDFT1) and 24-dehydrocholesterol reductase (DHCR24)], protein involved in transport of cholesterol (APOE, LDLr and ABCA1), the enzyme CYP46A1, responsible for the degradation of cholesterol, as well as proteins implicated in the regulation of cholesterol synthesis [sterol regulatory element-binding factor-2 (SREBF2), Liver X receptor type Beta (LXR beta)].

## Materials and Methods

### Animals

Twenty-four adult male Long-Evans rats weighing 300 g at the beginning of the experiment (Janvier Labs, France) were used in this study. After habituation to the animal facility, they were individually housed in a controlled environment under a 12-h light/dark cycle (lights on at 8 a.m.) with food available *ad libitum*. All experiments were conducted during the light phase and in accordance with the European Union directives (2010/63/EU) for the care of laboratory animals and approved by the local ethics committee (COMETHEA).

### Intermittent Exposure to Alcohol

Ethanol 96% (Cooper, France) was diluted with tap water to obtain a final concentration of 20% (v/v). Fluids were presented in glass bottles with stainless-steel drinking spouts inserted through two grommets in front of their home cage, according to a protocol similar to that used by Simms et al. (2008). Every second days, “alcohol” rats (*n* = 14) had the access for 24 h to two bottles, one containing the 20% ethanol solution or one containing tap water, allowing voluntary alcohol intake. During each exposure, the position of the ethanol bottle was changed to avoid side preferences. More precisely, on Mondays, Wednesdays and Fridays (10 a.m.), rats were given access to the two bottles, and the day after (10 a.m.), the two bottles were weighed and the bottle containing ethanol was replaced by a bottle containing water. Control rats (*n* = 10) had continuous access only to two bottles of water. We weighted the bottles and we measured alcohol intake (= g of pure ethanol/kg bodyweight/24 h), the preference for alcohol (= percentage of ethanol consumed over total fluid intake), to compare the volume of alcohol solution and water consumed, and the volume of fluid intake on “alcohol” and “water” days. The alternate exposure to alcohol lasted for 47 days, leading to 21 sessions of intermittent exposure. Animals were then exposed to two bottles of water for a 3-week abstinence period.

### Tissue Collection

Three weeks after the last exposure to ethanol, rats were anesthetized with pentobarbital (120 mg/kg, Dolethal^®^ Vetoquinol France), decapitated and brains were removed, and dissected using a rat matrix (WPI, London). Brain structures, namely the PFC, the NAc, the mesencephalon and the amygdala, were isolated by blunt dissection and stored at −80°C until used.

### RNA Isolation and Reverse Transcription

Total RNA was isolated using TRIzol Reagent and chloroform and then purified using Macherey Nagel kit (Macherey Nagel, France). The RNA concentration was determined using the NanoDrop ND-1000 spectrophotometer (Thermo Fisher Scientific, Waltham, MA, USA) and the RNA integrity was evaluated by the Agilent 2100 Bioanalyzer System (Agilent 2100 Bioanalyzer). All RNAs had a RIN (RNA integrity number) above eight.

### Reverse Transcription and Quantitative Real Time PCR (RT-PCR)

Equal amounts of RNA input (1 μg) were reverse-transcripted using Verso cDNA synthesis kit (Thermo Fisher Scientific, Waltham, MA, USA; Dutscher, France). Real time qPCR was performed using the LightCycler480 detection system (Roche, France). Reaction mix was exposed to the amplification program consisting in one cycle (95°C for 5 min), followed by 45 cycles, including three phases (phase 1: 95°C for 20 s; phase 2: 64°C for 15 s for all primers (except for hrpt1 = 60°C); phase 3: 72°C for 20 s).

The sequences of primers used (Supplementary Table S1) were either designed using Primer-BLAST software^1^, or found in previous publications. Results were normalized to three housekeeping genes (*gapdh, β-actin* and *hrpt1*) and reported as RNA fold changes according to the ΔΔCT method (2^ΔΔCT^ = 2^ΔCT sample − mean ΔCT “control animal”^).

### Statistical Analysis

Behavioral data were analyzed using one-way or two-way ANOVA for repeated measures followed by Newman–Keuls *post hoc*. For RT-PCR, we used a Student *t*-test to compare ethanol-exposed rats (*n* = 14) and control rats (*n* = 10). The null hypothesis was rejected at *p* < 0.05.

## Results

### Rats Show Escalation of Alcohol Intake and Preference Over Time

**Figure 1:**
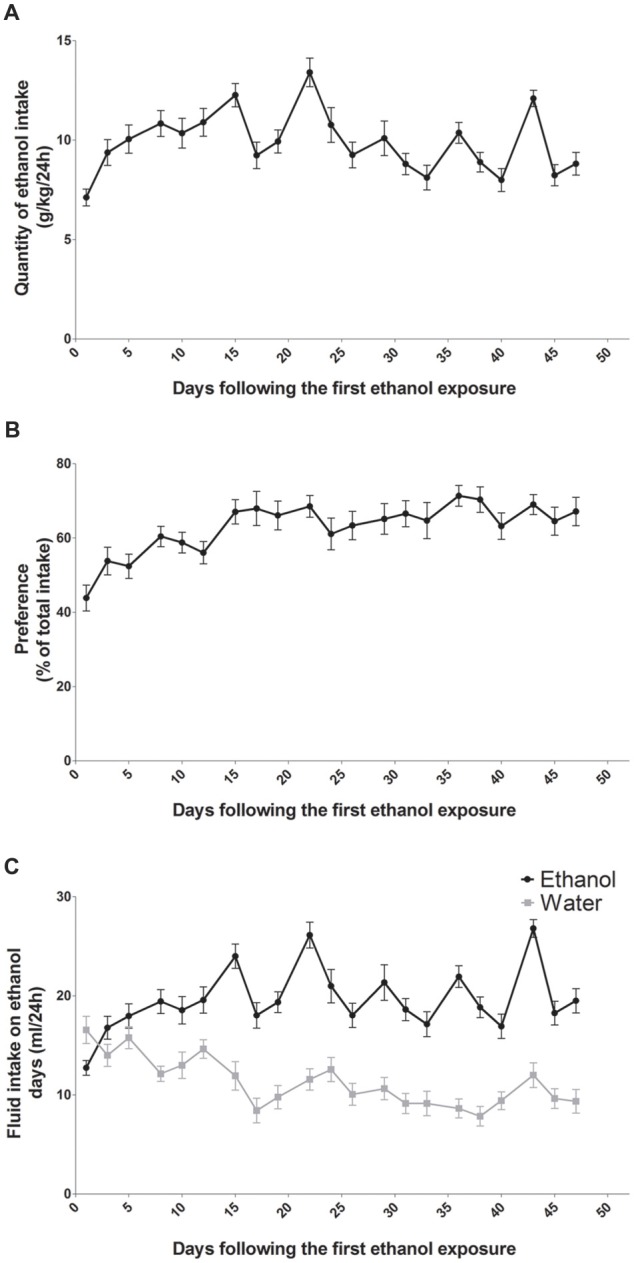
Alcohol intake and preference in rats exposed to an intermittent-access to ethanol. Ethanol intake **(A)** and preference for ethanol over water **(B)** in rat exposed to two bottle one containing water and one containing ethanol 20% (*n* = 14). Volumes of ethanol (black curve) and water (gray curve) consumed on alcohol days **(C)**.

In a two-bottle-choice procedure, intermittent-access to the 20% ethanol solution resulted in a steady increase in ethanol consumption (*n* = 14; Figure 1). Ethanol intake was 7 g/kg/24 h at the beginning and over time it reached a mean of 9 g/kg/24 h at the end of the study (Figure 1A). Statistical analysis revealed a significant effect of sessions for ethanol intake (*F*_(13,20)_ = 9.99, *p* < 0.0001). The preference for the bottle containing alcohol was aproximately 45% at the beginning and it reached 70% at the end of the study (Figure 1B). Statistical analysis revealed a significant effect of sessions for alcohol preference (*F*_(13,20)_ = 6.56, *p* < 0.0001). On the first day of exposure to alcohol, rats drank a mean of 12.5 ml/24 h of alcohol and a mean of 16 ml/24 h of water (Figure 1C). At the end of the study, rats drank a mean of 19 ml/24 h of alcohol and a mean of 11 ml/24 h of water (Figure 1C). Statistical analysis revealed a significant effect of sessions (*F*_(13,20)_ = 16.90, *p* < 0.0001), of alcohol (*F*_(1,13)_ = 26.54, *p* < 0.0001) and a significant sessions × alcohol interaction (*F*_(13,20)_ = 8.00, *p* < 0.0001). Fluid consumption (ml/24 h) of rats exposed to the two-bottle choice on alcohol and water days was similar and remained stable throughout the experiment (around 27 ml/24 h; data not shown).

### Intermittent Alcohol Intake Induces Persistent Modifications in the Expression of Genes Involved in the Metabolism of Cholesterol in the PFC

**Figure 2:**
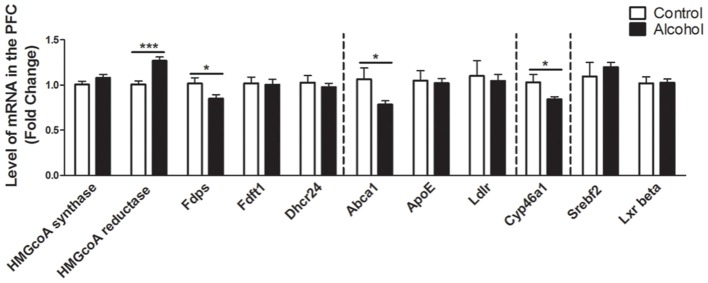
Changes in expression of genes involved in cholesterol metabolism in the PFC after exposure to alcohol. Expression of several genes involved in the metabolism of cholesterol in rats exposed to a two-bottle choice paradigm and water controls. Values represent the means ± SEM of 2^−ΔΔCT^ normalized to the control group. White bars correspond to control animals (*n* = 10) and black bars correspond to ethanol animals (*n* = 14). Student *t*-test, *p < 0.05, ***p < 0.001 different from control group. Abbreviations: Amy, amygdala; PFC, prefrontal cortex; HMGcoA, 3-hydroxyl-3-methylglutaryl-coenzyme A; Fdps, farnesyl diphosphate synthase; Fdft1, farnesyl-diphosphate farnesyltransferase 1; Dhcr24, 24-dehydrocholesterol reductase; Abca1, ATP-binding cassette A1; ApoE, apolipoprotein E; Ldlr, low density lipoprotein receptor; Cyp46a1, 24S cholesterol hydroxylase; Srebf2, sterol-regulatory element-binding factor; Lxr beta, liver X receptor type beta.

In the PFC (Figure 2), alcohol intake followed by 3 weeks of abstinence produced an increase in the expression of the gene encoding HMGCoA reductase, the rate limiting enzyme of cholesterol synthesis (+30%, *p* < 0.001) and a decrease in the expression of the gene encoding the enzyme FDPS, involved in the intermediate steps of cholesterol synthesis (−17%, *p* < 0.05) in rats exposed to alcohol (*n* = 14) compared to control group (*n* = 10). Alcohol consumption also produced a decrease in the expression of ABCA1 transporter, responsible for cholesterol export from cells (−20%, *p* < 0.05). In addition, alcohol intake produced a decrease in the expression of the enzyme CYP46A1, which degrades cholesterol (−15%, *p* < 0.05). All the other genes studied were not affected by exposure to alcohol.

### Intermittent Alcohol Intake Induces Persistent Modification in Genes Involved in the Metabolism of Cholesterol in the NAc

**Figure 3:**
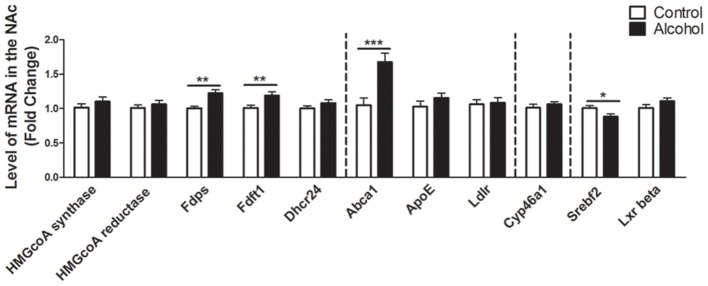
Changes in expression of genes involved in cholesterol metabolism in the NAc after exposure to alcohol. Expression of several genes involved in the metabolism of cholesterol in rats exposed to a two-bottle choice paradigm and water controls. Values represent the means ± SEM of 2^−ΔΔCT^ normalized to the control group. White bars correspond to control animals (*n* = 10) and black bars correspond to ethanol animals (*n* = 14). Student *t*-test, *p < 0.05, **p < 0.01, ***p < 0.001 different from control group. Abbreviations: NAc, nucleus accumbens; HMGcoA, 3-hydroxyl-3-methylglutaryl-coenzyme A; Fdps, farnesyl diphosphate synthase; Fdft1, farnesyl-diphosphate farnesyltransferase 1; Dhcr24, 24-dehydrocholesterol reductase; Abca1, ATP-binding cassette A1; ApoE, apolipoprotein E; Ldlr, low density lipoprotein receptor; Cyp46a1, 24S cholesterol hydroxylase; Srebf2, sterol-regulatory element-binding factor; Lxr beta, liver X receptor type beta.

In the NAc (Figure 3), alcohol intake followed by 3 weeks of abstinence produced an increase in the expression of the genes encoding FDPS and FDFT1, involved in the intermediate steps of cholesterol synthesis (+20%, *p* < 0.01 for FDPS and +20%, *p* < 0.01 for FDFT1) in rats exposed to alcohol (*n* = 14) compared to control group (*n* = 10). It also produced an increase in the expression of the gene encoding ABCA1 (+70%, *p* < 0.001). In addition, alcohol consumption produced a decrease in the expression of the gene encoding SREBF2 (−12%, *p* < 0.05). All the other genes studied were not affected by exposure to alcohol.

### Intermittent Alcohol Intake Induces Persistent Modification in Genes Involved in the Metabolism of Cholesterol in the Mesencephalon

**Figure 4:**
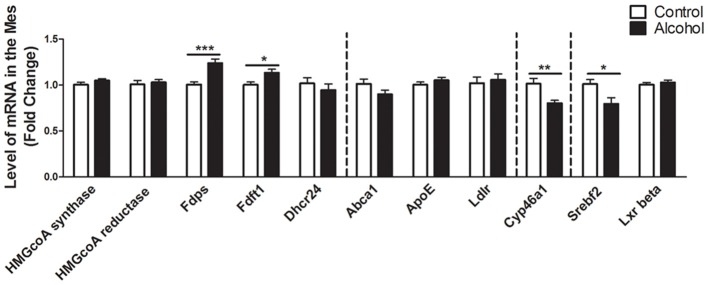
Changes in expression of genes involved in cholesterol metabolism in the mesencephalon after exposure to alcohol. Expression of several genes involved in the metabolism of cholesterol in rats exposed to a two-bottle choice paradigm and water controls. Values represent the means ± SEM of 2^−ΔΔCT^ normalized to the control group. White bars correspond to control animals (*n* = 10) and black bars correspond to ethanol animals (*n* = 14). Student *t*-test, *p < 0.05, **p < 0.01, ***p < 0.001 different from control group. Abbreviations: Mes, mesencephalon; HMGcoA, 3-hydroxyl-3-methylglutaryl-coenzyme A; Fdps, farnesyl diphosphate synthase; Fdft1, farnesyl-diphosphate farnesyltransferase 1; Dhcr24, 24-dehydrocholesterol reductase; Abca1, ATP-binding cassette A1; ApoE, apolipoprotein E; Ldlr, low density lipoprotein receptor; Cyp46a1, 24S cholesterol hydroxylase; Srebf2, sterol-regulatory element-binding factor; Lxr beta, liver X receptor type beta.

In the mesencephalon (Figure 4), alcohol intake followed by 3 weeks of abstinence caused an increase in the expression of the genes encoding FDPS and FDFT1, that are both involved in the cholesterol synthesis (+25%, *p* < 0.001 for FDPS and +15%, *p* < 0.05 for FDFT1) in rats exposed to alcohol (*n* = 14) compared to control group (*n* = 10). In addition, alcohol consumption produced a decrease in the expression of SREBF2 (−20%, *p* < 0.05). Alcohol intake also decreased the expression of CYP46A1 (−20%, *p* < 0.01). All the other genes studied were not affected by exposure to alcohol.

### Intermittent Alcohol Intake Induces Persistent Modification in Genes Involved in the Metabolism of Cholesterol in the Amygdala

**Figure 5:**
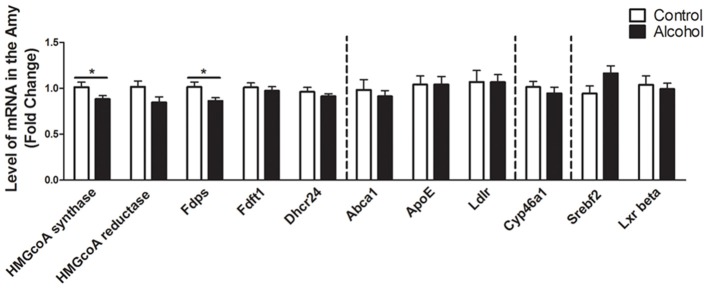
Changes in expression of genes involved in cholesterol metabolism in the amygdala after exposure to alcohol. Expression of several genes involved in the metabolism of cholesterol in rats exposed to a two-bottle choice paradigm and water controls. Values represent the means ± SEM of 2^−ΔΔCT^ normalized to the control group. White bars correspond to control animals (*n* = 10) and black bars correspond to ethanol animals (*n* = 14). Student *t*-test, *p < 0.05 different from control group. Abbreviations: Amy, amygdala; HMGcoA, 3-hydroxyl-3-methylglutaryl-coenzyme A; Fdps, farnesyl diphosphate synthase; Fdft1, farnesyl-diphosphate farnesyltransferase 1; Dhcr24, 24-dehydrocholesterol reductase; Abca1, ATP-binding cassette A1; ApoE, apolipoprotein E; Ldlr, low density lipoprotein receptor; Cyp46a1, 24S cholesterol hydroxylase; Srebf2, sterol-regulatory element-binding factor; Lxr beta, liver X receptor type beta.

In the amygdala (Figure 5), alcohol intake followed by 3 weeks of abstinence produced a decrease in the expression of the genes encoding HMGCoA synthase and FDPS (−10%, *p* < 0.05 for HMGCoA synthase and −15%, *p* < 0.05 for FDPS) in rats exposed to alcohol (*n* = 14) when compared to control rats (*n* = 10). All the other genes studied were not affected by exposure to alcohol.

## Discussion

In this study, we investigated the long-term changes in the expression of genes involved in cerebral cholesterol homeostasis induced by chronic alcohol exposure followed by a period of protracted abstinence. These changes might participate in the increased craving that appear during abstinence and in the consequent relapse to alcohol (Bienkowski et al., 2004; Pickens et al., 2011). For this, we allowed rats to drink alcohol intermittently for 47 days and, after a period of 3 weeks of forced abstinence, we measured the expression of genes encoding proteins involved in cholesterol metabolism in several brain areas involved in addiction processes. We found persistent modifications in the expression of several genes encoding proteins involved in the synthesis, transport and degradation of cholesterol in a region-specific manner. These changes might participate in the long-term adaptations associated with alcohol addiction and responsible for the high risks of relapse to this drug.

**Figure 6:**
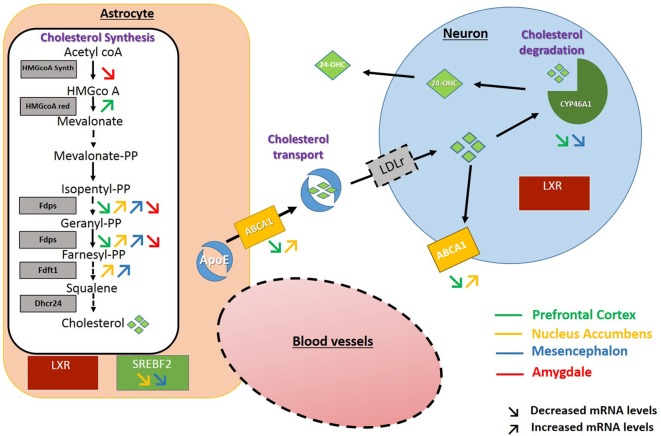
Schematic representation of the changes found in the expression of genes encoding proteins of the cholesterol metabolism. Increased and decreased expressions are represented by up and down arrows in the PFC (green arrows), NAc (yellow arrows), Mesencephalon (blue arrows), Amygdala (red arrows).

In the PFC, after 3 weeks of discontinuation of chronic intake of alcohol, we observed an increase in the expression of HMGCoA reductase, the rate limiting enzyme in the mevalonate pathway. In the NAc and the mesencephalon, the FDPS or FDFT1 enzymes, that act downstream in this pathway also showed increased expression levels. These results suggest that exposure to alcohol followed by 3 weeks of abstinence could result in increases in the production of cholesterol in these brain regions. In the NAc and the mesencephalon, we also found that alcohol exposure decreases the expression of SREBF2, a regulator of the transcription of genes encoding proteins of the mevalonate pathway. This effect could be a consequence of the increased levels of cholesterol triggered by the higher expression of enzymes responsible for its synthesis in these brain areas (Horton et al., 2003; Adams et al., 2004). Finally, in the PFC and the mesencephalon, we also observed a decrease in the expression of CYP46A1, the main enzyme involved in brain cholesterol catabolism, which could also lead to accumulation of cholesterol in these brain regions. Conversely in the amygdala, we mainly found a decreased expression of the enzyme HMGCoA synthase and FDPS involved in cholesterol synthesis, which suggests that alcohol exposure followed by 3 weeks of abstinence could lead to long-lasting decreases in cholesterol levels in this brain area. All the changes observed have been summarized in Figure 6.

Exposure to alcohol has been shown to lead to a great number of neuroadaptations. Some of these changes appear rapidly after the interruption of the alcohol intake and disappear within a few days (for review see Heilig et al., 2010; Roberto and Varodayan, 2017). Other adaptations could persist long after discontinuation of alcohol intake (Heilig et al., 2010) and be responsible for the incubation of craving that has been described after abstinence from alcohol consumption (Bienkowski et al., 2004; Pickens et al., 2011). Our study mainly focused on these changes because they are thought to play a critical role in relapse to alcohol use (Gilpin and Koob, 2008; Spanagel, 2009). Changes in the cerebral metabolism of cholesterol could participate in these neuroadaptations. For example, cholesterol could affect synaptic communication at the presynaptic level by modulating neurotransmitter uptake or release and also at the post-synaptic level by acting on the quantity of receptors expressed at the membrane. Concerning dopamine neurotransmission, using super-resolution microscopy, it has been shown that the dopaminergic transporter (DAT) may be sequestrated in cholesterol-dependent nanodomains in the membrane of presynaptic neurons and that cholesterol depletion reduces localization of the DAT in these nanodomains (Rahbek-Clemmensen et al., 2017). Other evidences suggest that cholesterol dysregulation may alter the DAT functioning (Jones et al., 2012; Luessen and Chen, 2016; Zeppelin et al., 2018). Thus, it is possible that if cholesterol homeostasis is dysregulated, the functioning of the dopamine system and eventually the response to future exposure to drugs may be changed, which may in turn increase the risks of addiction. Indeed, the activity of the dopaminergic neurotransmission was shown to be decreased after acute withdrawal (Carroll et al., 2006; Czoty, 2015; Karkhanis et al., 2015) and increased after protracted abstinence from alcohol (Hirth et al., 2016). Interestingly, changes in cholesterol content that might result from the changes we observed could participate in these effects observed after alcohol withdrawal. In addition, cholesterol homeostasis can participate in addiction processes by altering synaptic plasticity. For example, in culture of hippocampal neurons, cholesterol depletion by use of inhibitors of the HMGCoA reductase has been shown to impair synaptic vesicles exocytosis (Linetti et al., 2010) and, in cortical synaptosomes, cholesterol removal from the plasma membrane increases spontaneous glutamate release and reduced the evoked release (Teixeira et al., 2012). Also, in the hippocampus, activation of post-synaptic NMDARs during LTP is associated with a reduction in intracellular cholesterol content in neurons, which seems to facilitate the redistribution of AMPA receptor to the membrane (Brachet et al., 2015) and dietary cholesterol has been shown to affect synaptic plasticity (Wang and Zheng, 2015). Finally, endogenous 24S-hydroxycholesterol, the main metabolite of cholesterol in the brain, has been shown to modulate NMDAR-mediated function (Sun et al., 2016a). Altogether, these studies indicate that cholesterol may play a role in modulating neurotransmission and that changes in cholesterol metabolism induced by alcohol exposure followed by a 3-week period of abstinence reported in our study may participate in such neuroadaptations and in the long-term effects of alcohol that drive the addiction cycle (Koob and Le Moal, 2001; Sommer and Spanagel, 2013).

A few studies investigated the effects of alcohol exposure, followed or not by a withdrawal periods, on the expression of genes involved in cholesterol metabolism both in humans post-mortem tissues (Lewohl et al., 2000; Mayfield et al., 2002; Flatscher-Bader et al., 2008, 2010) and in animal models (for example see McBride et al., 2013; McClintick et al., 2016). Some of these studies targeted the same brain areas we investigated, namely the NAc (Bell et al., 2009), the ventral tegmental area (McBride et al., 2013), the amygdala (McBride et al., 2014) or the PFC (McClintick et al., 2018), and some have focused on other structures such as the dorsal raphe nucleus (McClintick et al., 2015) or the periaqueductal gray (McClintick et al., 2016). These studies mostly used large-scale transcriptomic analysis and some of them, but not all, also found that cerebral cholesterol metabolism is affected by alcohol consumption. Interestingly, some of the alterations reported in these studies are similar to the ones found in our study. For example, McBride et al. (2013) observed a decreased expression of SREBF1 in the ventral tegmental area of alcohol-preferring (P) rats following repeated excessive binge-like alcohol drinking followed by protracted abstinence, an effect similar to what we observed in the mesencephalon for SREBF2. In the periaqueductal gray, binge-like alcohol consumption produced a rapid decrease in the expression of the HMGCoA reductase and synthase, FDFT1 and FDPS (McClintick et al., 2016). These results (even if they were obtained after acute withdrawal from alcohol) are similar to those we found in the amygdala. Interestingly, these two structures both control emotional processes like fear and anxiety and interact strongly with each other (Graeff et al., 1993; Johansen et al., 2010). It is to note that, the expression of the HMGCoA reductase and SREBF2 genes were also decreased in the ventral hippocampus, also involved in anxiety-like behavior, of adolescent rat exposed to alcohol and sacrificed rapidly at the end of the exposure to alcohol (McClintick et al., 2018). It could be speculated that the transcriptional changes we observed, if they are translated in protein changes and modulation of cholesterol levels, can contribute to functional plasticity and, consequently, to the long-term increase in anxiety-like behavior following chronic ethanol exposure (Läck et al., 2005; Gilpin et al., 2015).

It is to note that some discrepancies also exist between our results and those previously reported. The differences between these studies and ours could be due to several methodological differences such as animal species used (mice vs. rats), the genetic background of rats used (selected alcohol-preferring vs. Long-Evans), the type of alcohol exposure (administered by the experimenter, forced or voluntary intake), the age of the rats (adolescence vs. adulthood), the sensitivity of the approach used to evaluate the gene expression (qPCR vs. large-scale analysis) and also the time of abstinence (from no abstinence to short-term and long-term abstinence). In fact, acute withdrawal from alcohol has been shown to produce behavioral and neurobiological effects that are the consequence of homeostatic adaptation associated with chronic exposure to alcohol (Koob and Le Moal, 2001; Sommer and Spanagel, 2013). On the other hand, the behavioral effects and most of the neurobiological effects disappear within a few days whereas some may persist and other may develop over time (Heilig et al., 2010). Interestingly, exposure to alcohol was reported to produce little changes in gene expression in the NAc in animal sacrificed immediately after the end of ethanol exposure (Morud et al., 2017), when changes have been described after protracted abstinence (Hirth et al., 2016). Importantly, brain cholesterol homeostasis is a very tightly regulated phenomenon and several redundant mechanisms exist to maintain cholesterol levels within the appropriated physiological range. Therefore, it is possible that the brain could find several different strategies to react to changes in cholesterol levels associated with drug and alcohol use and that, conversely, changes in different proteins involved in cholesterol homeostasis could ultimately produce similar changes in cholesterol levels.

It is important to notice that this is an exploratory study aiming at looking for evidence that alcohol may produce neuroadaptations in cholesterol homeostasis in regions involved in addiction processes. For this, we chose to focus on changes in gene expression as measured by RT-PCR techniques, which allows screening a large number of genes in several brain areas. On the other hand, this study does not provide direct information concerning the levels of proteins that are encoded by those genes and the levels of cholesterol, its precursors and its metabolites. Future studies are needed to determine the exact changes produced by chronic voluntary alcohol exposure and their functional consequences. Notwithstanding these limitations our study clearly show that the cholesterol system reacts to chronic administration of alcohol and that neurobiological traces of alcohol exposure could be found in this system even after several weeks of discontinuation of alcohol intake, a period when craving for alcohol and risks of relapse are high (Bienkowski et al., 2004; Pickens et al., 2011).

## Conclusion

In conclusion, in this explorative study, we show that voluntary intake of alcohol followed by protracted abstinence produces long-lasting changes in the expression of genes involved in cholesterol homeostasis in brain regions involved in addiction. Although further investigation is needed to determine the functional consequences of these alterations our results suggest that cholesterol homeostasis may be involved in development and maintenance of addiction to alcohol.

## Author Contributions

MS and NT designed the general experiments, analyzed the data and wrote the article with contribution by JA. JA and ED conducted the experiments. All authors contributed to the critical revision of the data and of the final version of the manuscript.

## Conflict of Interest Statement

The authors declare that the research was conducted in the absence of any commercial or financial relationships that could be construed as a potential conflict of interest.

## Footnotes

^1^http://www.ncbi.nlm.nih.gov/tools/primer-blast/index.cgi?LINK_LOC=BlastHome

## Supplementary Material

TABLE S1 Primer sequences used for RT-qPCR (for, forward primers; rev, reverse primers).
