# Supplementary material for: Persistent Neuroadaptations in the Expression of Genes Involved in Cholesterol Homeostasis Induced by Chronic, Voluntary Alcohol Intake in Rats
Source: Front Mol Neurosci. 2018 Dec 13;11:457. doi: 10.3389/fnmol.2018.00457 (PMC6300585; doi:10.3389/fnmol.2018.00457)
Supplement: TABLE S1 — Primer sequences used for RT-qPCR (for, forward primers; rev, reverse primers). [file Table_1.pdf]

Genes	Accession number	Primers	Ref
HMGCoA synthase *Hydroxymethylglutaryl-CoA synthase*	NM_017268.1	For :ATCGCGTTTGGTGCCTGAAG<br>Rev: AAGGGCAACGATTCCCACAT	
HMGCoA reductase *Hydroxymethylglutaryl-CoA reductase*	NM_013134.2	For: GGTGCATCGCCATCCTGTAC<br>Rev: GCTGACGCAGGTTCTGGAA	
Fdft1 *Farnesyl diphosphate farnesyl transferase*	NM_019238.2	For: ACCAACGCCCTACAACACAT<br>Rev: GTAACTGCTTGCCCCTTCC	A
Fdps *Farnesyl diphosphate synthase*	NM_031840.1	For: TCAGTGTCTGCTACGAGCC<br>Rev: TCGTACTTGAAGAACACACTCC	B
Abca1 *ATP binding cassette A1*	NM_178095.2	For: GGTAGTGTGGCCACTTTCGT<br>Rev: TCTGGGCCTGATGAAAAATC	C
ApoE *Apolipoprotein E*	J00705	For: TCCATTGCCTCCACCACAGT<br>Rev: GGGCGTAGGTGAGGGATGA	
Ldlr *Low Density lipoprotein receptor*	NM_175762.2	For: TTCTTCAGGTTGGGGATCAG<br>Rev: CAGCTCTGTGTGAACCTGGA	C
Dhcr24 *24-Dehydrocholesterol reductase*	NM_001080148.1	For: TAGAGCCCAGCAAGCTGAAT<br>Rev: ATCCAGCCAAAGAGGTAGCG	
CYP46A1 *24S cholesterol hydroxylase*	NM_001108723.1	For: GTGCCACCATCGACATCCTG<br>Rev: GGTGTTACGGGACGCACTGATAC	D
Srebf2 *Sterol regulatory element-binding protein-2*	NM_001033694.1	For: GTCCTCACCTTCCTGGGTCT<br>Rev: CAGCAGTAGAGTCGGCATCA	A
Lxr Beta *Liver X receptor beta*	NM_031626.1	For: TGAAGGCATCCACCATCGAG<br>Rev: CGTGATGCACTCTGTCTCGT	
Gapdh *Glyceraldehyde-3-Phosphate Dehydrogenase*	NM_017008.4	For: ATGGGAAGGTCGGTGTGAAC<br>Rev: ACTCCACGACATACTCAGCAC	
ActB *Actin beta*	NM_031144.3	For: GGAGAAGATTTGGCACCAC<br>Rev: AGGCATACAGGGACAACAC	
Hprt1 *Hypoxanthine Phosphoribosyltransferase 1*	NM_012583.2	For: TGGTCAAGCAGTACAGCCCC<br>Rev: TAGTGGCCACATCAACAGGA	

Table : Primer sequences used for RT-qPCR (For: Forward primers; Rev: Reverse primers)

References used:

A. Caimari, A., Oliver, P., Rodenburg, W., Keijer, J., and Palou, A. (2010). Feeding conditions control the expression of genes involved in sterol metabolism in peripheral blood mononuclear cells of normoweight and diet-induced (cafeteria) obese rats. *J. Nutr. Biochem.* 21, 1127–1133. doi:10.1016/j.jnutbio.2009.10.001.

B. Yuan, F., Wang, H., Tian, Y., Li, Q., He, L., Li, N., et al. (2016). Fish oil alleviated high-fat diet-induced non-alcoholic fatty liver disease via regulating hepatic lipids metabolism and metaflammation: A transcriptomic study. *Lipids Health Dis.* 15. doi:10.1186/s12944-016-0190-y.

C. Fernández-Pérez, L., Santana-Farré, R., De Mirecki-Garrido, M., García, I., Guerra, B., Mateo-Díaz, C., et al. (2014). Lipid profiling and transcriptomic analysis reveals a functional interplay between estradiol and growth hormone in liver. *PLoS One* 9. doi:10.1371/journal.pone.0096305.

D. Ishikawa, M., Yoshitomi, T., Zorumski, C. F., and Izumi, Y. (2016). 24(S)-Hydroxycholesterol protects the ex vivo rat retina from injury by elevated hydrostatic pressure. *Sci. Rep.* 6. doi:10.1038/srep33886.
